# Sunshine vitamin and thyroid

**DOI:** 10.1007/s11154-017-9406-3

**Published:** 2017-01-14

**Authors:** Immacolata Cristina Nettore, Luigi Albano, Paola Ungaro, Annamaria Colao, Paolo Emidio Macchia

**Affiliations:** 1 Dipartimento di Medicina Clinica e Chirurgia, Università degli Studi di Napoli “Federico II”, Via S. Pansini, 5, 80131 Napoli, Italy; 2 Dipartimento di Scienze Mediche Traslazionali, Università degli Studi di Napoli “Federico II”, Napoli, Italy; 3 Istituto di Endocrinologia ed Oncologia Sperimentale del CNR (IEOS-CNR) “G. Salvatore”, Napoli, Italy

**Keywords:** Vitamin D, Hashimoto’s thyroiditis, Graves’ disease, Post-partum thyroiditis, Thyroid cancer

## Abstract

Vitamin D exerts its canonical roles on the musculoskeletal system and in the calcium/phosphorus homeostasis. In the last years, increasing evidences suggested several extra-skeletal actions of this hormone, indicating that vitamin D may produce effects in almost all the body tissues. These are mediated by the presence of vitamin D receptor (VDR) and thanks to the presence of the 1-α-hydroxylase, the protein that converts the 25-hydroxyvitamin (calcidiol) to the active form 1,25-dihydroxyvitamin (calcitriol). Several studies evaluated the possible role of vitamin D in the pathogenesis of thyroid diseases, and this review will focus on the available data of the literature evaluating the association between vitamin D and thyroid function, vitamin D and autoimmune thyroid diseases, including Hashimoto’s thyroiditis, Graves’ disease and post-partum thyroiditis, and vitamin D and thyroid cancer.

## Introduction

The term ‘vitamin D’ generally indicates two different compounds, the cholecalciferol (or vitamin D3) and the ergocalciferol (vitamin D2). Vitamin D3 is normally synthetized in the skin upon exposure to ultraviolet B (UVB) radiation by the action of the 7-dehydrocholesterol reductase. In addition, it can be introduced with the diet from few dietary sources (i.e. fatty fish). Ergocalciferol represents the dietary source of vitamin D and it is synthesized by plants and fungi. Both forms are transferred to the liver, were they are hydroxylated to 25-hydroxyvitamin D (25-OH-D3, or calcidiol). This is the major circulating and storage form of vitamin D [1]. Evaluation of serum 25-(OH)-D3 is considered to provide a reliable evaluation of the vitamin D status [2].

The vitamin D active form is produced by the 1-α-hydroxylase protein. This protein, encoded by the CYP27B1 gene and expressed mainly in the kidney, determines the hydroxylation of calcidiol to 1,25-(OH)_2_D3 (calcitriol). Calcitriol formation is down-regulated via a negative feedback by calcitriol concentrations and by the fibroblast growth factor 23 (FGF23). Calctriol is inactivated by the action of the 24-hydroxylase.

The active form of vitamin D binds nuclear vitamin D receptor (VDR) and heterodimerizes with retinoic acid. This complex interacts with vitamin D responsive elements of target genes to exert its effects. Also, a form of a membrane-bound vitamin D receptor has been hypothesized, which would mediate non-genomic, rapid effects of calcitriol [3].

Several polymorphic variants of the genes involved in metabolism, transport, and activity of vitamin D have been described in the last years. The best characterized are the four single-nucleotide polymorphic (SNP) variants of the VDR gene (ApaI, BsmI, FokI, and TaqI) that have been associated with several pathological situations, including autoimmune disorders or cancers [4, 5].

Other genes, whose variants may lead to altered availability and metabolism of vitamin D are: DHCR7, GC, CYP2R1, CYP27B1, CYP24A1. These genes encode for proteins mentioned above: 7-dehydrocholesterol reductase, vitamin D binding protein (DBP), 25-hydroxylase, 1-alpha-hydroxylase, and 24-hydroxylase, respectively.

In the last years, the idea that Vitamin D is an hormone that acts not only on the skeletal system but on a great number of target tissues has been supported by the identification of the VDR in nearly all tissue types [6], including the thyroid gland [1, 7].

Herein the relationships between vitamin D and thyroid diseases have been reviewed. The manuscript is based on an electronic literature search of PubMed database performed in September and October 2016. The selection of the articles was done using the subsequent search terms in association with vitamin D: Hashimoto’s thyroiditis; Graves’ disease; Post-partum thyroiditis; thyroid cancer.

## Vitamin D and thyroid status

Thyroid hormones (thyroxine or T4 and triiodothyronine or T3, TH) are vital for the control of metabolism and for maintaining specific function of several tissue and cell types. Biosynthesis of TH occurs within the thyroid gland and it is stimulated by the thyroid-stimulating hormone (TSH) secreted by the pituitary. Data on the direct interactions between Vitamin D and circulating TH or TSH are very poor.

Experiments with rodents demonstrated that in rats the administration of calcitriol (0.05 microgram/kg per day for 3 days) did not change TSH or T4 [8]. By contrast, rats fed with a severely vitamin D deficient diet had lower serum TSH but T4 levels similar to vitamin D sufficient animals [9]. In addition, VDR knock out mice had no alterations in morphology and function of the thyroid gland and showed only a slight reduction in circulating TSH levels [10].

In healthy humans, no clear data are available on the effects of vitamin D status (excess or deficiency) on thyroid function.

In a cohort of hospitalized patients without history of thyroid disease, no differences were observed in basal serum TSH between patients with a very low (≤10 ng/mL) or high (≥40 ng/mL) vitamin D status [11]. Other reports indicate contrasting results. Chailurkit and coworkers studied a Thai cohort and demonstrated that high vitamin D status is associated with low circulating thyrotropin [12] only in young, while Zang and others reported a similar association also in elderly [13].

Mackawy et al. suggested that hypothyroidism is associated to low vitamin D levels with a negative correlation between TSH and calcidiol [14]. In contrast, Bouillon and coworkers found that vitamin D levels had no differences between hypothyroid or hyperthyroid patients and healthy subjects [15]. Finally, no effects of hyperthyroidism on circulating Vitamin D levels have also been reported by both Jastrup and Macfarlane [16, 17].

## Vitamin D and autoimmune thyroid disorders

Several studies indicated that vitamin D plays a significant role in the modulation of the immune system [18, 19]. Immune cells express both VDR and the 1-a-hydroxylase which is responsible for 25-hydroxyvitamin D activation. Indeed, vitamin D has important effects on both monocytes and dendritic cells (DC) including inhibition of inflammatory cytokines (interleukin (IL)-1, IL-6, IL-8, IL-12 and tumor necrosis factor (TNF)-α) in monocyte and reduction of Major Histocompatibility Complex (MHC) class II molecules expression. Moreover, vitamin D also determines suppression of T cell proliferation [20], whose final effect is a reduction in the number of antigen-presenting cells. Globally, vitamin D may enhance the innate immune system and regulate the adaptive immune system, promoting immune tolerance and acting to decrease the likelihood of developing autoimmune disease [18].

Autoimmune thyroid diseases (AITD) are the most frequent autoimmune disorders, and the most common pathological conditions of the thyroid gland occurring in approximately 5% of the population [21, 22]. The AITD comprise two main clinical presentations: Graves’ disease (GD) and Hashimoto’s thyroiditis (HT). Both forms are characterized by lymphocytic infiltration of the thyroid parenchyma, but while GD is clinically characterized by hyperthyroidism, ophthalmopathy and pretibial myxedema [23], the clinical hallmarks of HT is the hypothyroidism, determined by lymphocytic destruction of the thyroid gland [24].

As the majority of autoimmune disorders, AITDs is the consequence of a complex interaction between genetic susceptibility factors (i.e. thyroid-specific and immunoregulatory genes), existential factors (sex, parity, etc.), and various environmental triggers (i.e. cigarette smoking, stress, iodine, selenium, etc.) [25].

The role of both vitamin D and VDR in the pathogenesis of AITD have largely been investigated in the last years. Vitamin D receptor is expressed in lymphocytes, macrophages as well as in antigen presenting cells [26]. The innate immune system is activated in presence of Vitamin D, while the acquired immune response is inhibited. Moreover, associations between autoimmune disease and reduction of Vitamin D circulating levels have recently been reviewed [20]. With a specific focus on autoimmune thyroid disorders, several observations have been published in both animal models and in human studies.

### Animal models

Mice previously sensitized with porcine thyroglobulin have been intraperitoneal injected with or without calcitriol (0.1–0.2 micrograms per kg body weight daily) by Fournier and coworkers. Animals receiving these suboptimal doses of vitamin D presented a reduction in the severity thyroid inflammation compared to placebo treated mice [27]. The effects were even higher when mice were treated with both calcitriol and cyclosporine [28].

Liu and coworkers pretreated mice with intraperitoneal injection of calcitriol (5 micrograms per kg every 48 h) before performing sensitization with porcine thyroglobulin. Contrary to what observed in the placebo group, the thyroid not showed the typical inflammation signs, suggesting a protective role of vitamin D in prevention of thyroiditis [29].

Effects of vitamin D in GD animal models have been studied by Misharin et al. [30]. BALB/c mice have been produced as model of GD by immunization with adenovirus encoding the A-subunit of thyrotropin receptor. Compared with mice fed regular chow, hyperthyroid BALB/mice fed with a vitamin D deprived diet showed fewer splenic B cells, decreased interferon-gamma responses to mitogen and lack of memory T-cell responses to A-subunit protein, but no differences in TSHR antibody levels have been observed. Moreover, vitamin D-deficient BALB/c mice had lower preimmunization T_4_ levels and developed persistent hyperthyroidism suggesting that vitamin D directly modulates thyroid function in this animal model [30].

### Human studies

In the last years, several studies have investigated the circulating vitamin D levels in patients with AITD. A weak connection between low vitamin D levels and AITDs was identified in a study conducted in a population from India [31], while no correlation between vitamin D and Ab-Tg antibodies was demonstrated in Thai subjects [12].

By contrast, Kivity et al. observed that anti-thyroid antibodies were more frequently elevated in patients with vitamin D deficiency [32]. The same study, however, indicates that the prevalence of vitamin D deficiency was similar between hypothyroid patients with AITDs or without AITD (72% vs 52%, *p* = 0.08), not allowing to exclude that vitamin D deficiency is determined by hypothyroidism and not a primary phenomenon involved in AITD pathogenesis.

Tamer et al. demonstrated that patients with HT had lower vitamin D levels when compared to age- and sex-matched controls. Moreover, vitamin D insufficiency (<30 ng/mL) occurred more frequently in patients with HT rather than in a healthy population [33]. Nevertheless, the authors were not able to demonstrate a significant difference among the degree of vitamin D insufficiency between hypothyroid, euthyroid or hyperthyroid patients with HT, suggesting that vitamin D levels do not correlate with the progress of damage to thyrocyte. In contrast, a potential role of vitamin D in the development or progression of HT has been suggested by Bozkurt et al., demonstrating a correlation between severity of vitamin D deficiency and duration of HT, antibody levels and thyroid volume [34].

The association between AITD and calcidiol was also investigated by Choi and coworkers in a large cross-sectional study, involving about 6700 participants. The authors demonstrated that the levels of serum vitamin D were significantly lower in pre-menopausal, but not in post-menopausal women with AITD [35].

Shin et al. reported that patients with elevated anti-thyroid antibodies had significantly lower levels of serum 25(OH)D3 when compared to normal subjects. Moreover, after adjusting for age, sex, and body mass index, a negative correlation (*r* = −0.252, *p* < 0.001) was recognized between 25(OH)D3 and TPOAb levels in the AITDs patients [36].

By comparing newly diagnosed AITD patients with healthy age-matched controls, Unal et al. demonstrated that both HT and GD patients had lower circulating 25-OH-D3 compared to controls [37].

The relationships between vitamin D levels and HT in children have been investigated by Camurdan et al. The authors observed lower vitamin D levels and higher prevalence of vitamin D deficiency in children with new diagnosis of HT compared to sex- and age-matched controls [38]. Relation between vitamin D and AITD in young was recently studied also in a cohort of 56 Egyptian children with AITD and 56 healthy controls [39]. Also this report indicates that vitamin D deficiency is more frequent in the AITD group compared to the control subjects, and a significant negative correlations can be demonstrated between serum 25-OH vitamin D and age, duration of the disease, BMI, anti-TPO and anti-Tg antibodies and TSH. On the basis of these observations, the authors supposed that the vitamin D level is not an independent risk for the progression of AITD to overt hypothyroidism [39]. Sönmezgöz et al. confirmed these results in a group of 136 Turkish children. The prevalence of vitamin D deficiency was higher (76%) in HT patients than in the control group (35%). All hypothyroid HT patients also had a vitamin D deficiency [40]. Similar results have also been reported by Evliyaoğlu, who measured serum 25-OH vitamin D3 levels in 169 subjects, demonstrating that levels lower than 20 ng/mL were associated to HT in children and adolescents [41].

The relationships between vitamin D and GD have been less investigated. Kivity et al. reported significantly higher prevalence of vitamin D deficiency in GD patients than in healthy individuals matched by age (64% vs 30% respectively, *p* < 0.01) [32]. Newly identified GD female patients were studied for vitamin D levels by Yasuda et al. in 2012. The authors reported a decrease in the circulating vitamin D levels in GD patients and demonstrated a significant association of vitamin D deficiency with thyroid volume, but not with TRAb levels or thyroid function [42]. The same authors also found lower vitamin D levels in female GD patients without remission than in those with remission [42].

All the data on the associations between vitamin D and GD have been reviewed in a recent meta-analysis by Xu and coworkers. In this report, the authors conclude that low vitamin D status may increase the risk of Graves’ disease, however pathogenetic mechanisms related to this association still remains unclear, and it has not yet been clarified if supplement of vitamin D may have beneficial effect in GD [43].

To date, several authors have studied the association between functional polymorphism in the VDR gene and AITD risk. The VDR is the specific vitamin D receptor and its activity can be compromised by certain polymorphisms. The first meta-analysis performed to assess the association between the alleles of vitamin D receptor gene polymorphisms and Graves’ disease was performed by Zhou and coworkers in 2008. The authors concluded that ApaI, BsmI and FokI polymorphisms of the VDR gene were associated with susceptibility to GD in Asian populations, while ApaI, BsmI, TaqI and FokI polymorphisms were not associated with GD in Caucasian populations [44]. In 2013, Feng and coworkers performed a meta-analysis demonstrating a relevant link between VDR polymorphism and autoimmune thyroiditis [45]. The results indicated that the BsmI (rs1544410) or TaqI (rs731236) polymorphisms were significantly associated with AITD risk, while ApaI (rs7975232) or FokI (rs2228570) polymorphisms were not. Later, it has been demonstrated that the frequency of allele TT for the TaqI was higher in GD patients rather than in HT patients, while the frequency of the C allele for the ApaI was higher in GD patients than in normal controls [46]. Meng et al. [47] also demonstrated a higher frequency of allele A in ApaI in GD patients compared to controls, but no significant difference were found in BsmI, FokI and TaqI polymorphisms.

In conclusion, despite the number of papers studying the associations between vitamin D and AITD is constantly increasing, data are still not conclusive. There are suggestions indicating that vitamin D deficiency may be a condition associated with a higher risk of developing AITD, but it is still unclear whether this has a specific role in the pathogenesis of the diseases or is a consequence of the disease. Moreover, it has not been defined if vitamin D supplementation may modulate the evolution or the treatments of AITD.

## Vitamin D and post-partum thyroiditis

Postpartum thyroiditis is a form of subacute thyroiditis that normally occurs within 6–12 months after delivery. The prevalence of postpartum thyroiditis ranges from 1.1 to 16.7% of all pregnancies, with an overall incidence of 7.5% [48]. As most of the subacute thyroiditis the clinical course is characterized by a thyrotoxic phase occurring 1–3 months after parturition, followed by hypothyroidism at 3–6 months after delivery [49]. Finally, normal thyroid function is usually achieved within a year, however about 25% of women with a history of PPT will develop permanent hypothyroidism in the ensuing 10 years [50].

Several papers have recently analyzed the association between circulating levels of vitamin D and postpartum thyroiditis (PPT).

Krysiak and coworkers investigated the levels of 25-hydroxyvitamin D and parathyroid hormone in four groups of non-lactating women who gave birth within 12 months before the beginning of the study: group A was composed by hypothyroid women with post-partum thyroiditis (*n* = 14), group B by euthyroid females with post-partum thyroiditis (*n* = 14); group C by women with non-autoimmune hypothyroidism (*n* = 16) and group D by healthy euthyroid females (*n* = 15). Serum levels of 25-hydroxyvitamin D were lower and PTH levels were higher in patients with post-partum thyroiditis than in patients without thyroid autoimmunity. L-thyroxine treatment increased 25-hydroxyvitamin D and reduced PTH levels only in hypothyroid women with post-partum thyroiditis. The results suggested an association between vitamin D status and post-partum thyroiditis, despite the authors had to admit several limitations of the study [51].

More recently, the same group has studied 38 non-lactating L-thyroxine-treated women with postpartum thyroiditis (PPT) and 21 matched healthy postpartum women. 25-hydroxy vitamin D levels were lower in women with PPT than in healthy women, and anti-TPO and anti-Tg antibody titers inversely correlated with vitamin D status. The authors noted that vitamin D supplementation was able to reduce titers of thyroid peroxidase and suggested that vitamin D supplementation may bring benefits to L-thyroxine-treated women with PPT [52]. This work, however, received strong critics in a letter in the same issue of the journal [53] and data on the role of vitamin D in PPT, in our view, are still incomplete and very controversial.

## Vitamin D and thyroid cancer

In addition to its main role in calcium homeostasis, vitamin D has been associated with risk for several types of cancer, probably in consequence of its effects on cell proliferation, differentiation, apoptosis, and anti-angiogenesis [54, 55]. Several studies suggest that vitamin D may play a role in the pathogenesis, progression, and therapy for cancer. It has been suggested that lower serum vitamin D levels can be associated with higher risk of cancers. In this view, suboptimal vitamin D concentrations in serum have been proposed as an important cancer risk factor for several types of tumors, including thyroid cancers [3, 56].

To date, very limited data are available on the clinical significance of vitamin D supplementation as measure to reduce the risk of cancer. Many *in vitro* and animal studies indicate that high concentrations vitamin can reduce the progression of cell cycle, may induces apoptosis and slow neoplastic cell growth. Moreover, vitamin D has been also demonstrated to potentiates the antitumor activity of various anticancer drugs, however the mechanisms that underlie the antitumor activities of vitamin D are still unclear. In view additional studies are required in order to better define the role of vitamin D in cancer, and this is true also for thyroid cancers [57]. Here, the effects of vitamin D potential role in thyroid cancers will be reviewed, considering the *in vitro*, *in vivo* and human available data.

### *In vitro* evidences

The vitamin D activity is mediated by binding to VDR, which is expressed in both normal and malignant epithelial thyroid cells. Both VDR and 1-α-hydroxylase were found to be increased in papillary thyroid carcinoma (PTC) compared with normal thyroid tissue [58]. Moreover, VDR expression was reduced in metastatic lymph nodes of PTC compared with both normal thyroid tissue and primary PTC, suggesting that vitamin D pathway may be associated with differentiation and reduced proliferation in PTC [58]. Additional *in vitro* studies demonstrated that 25(OH)D3 was able to decrease proliferative activity of follicular thyroid cells [59] and modulate expression of ECM protein-1 (ECM1) and the type II transmembrane serine protease-4 (TMPRSS4), two independent predictor of thyroid carcinoma [60]. Indeed, RT-PCR experiments revealed a higher mRNA expression of ECM1, TMPRSS4 and VDR in the malignant thyroid tissues compared to the normal thyroid tissues from the same patients. ECM1 has been detected in numerous malignant epithelial tumors, while TMPRSS4 is a protein involved in invasion, metastasis, migration, adhesion, and epithelial-mesenchymal transition in cancer cells. Therefore, the association between VDR to ECM1 and TMPRSS4, suggested a potential role of VDR in thyroid carcinoma [60].

The study by Clickspoor and coworkers demonstrated an increased expression of VDR, CYP24A1 and CYP27B1 genes, all key players involved in local vitamin D signaling, in benign and differentiated malignant thyroid tumors. A decrease in these genes expression was observed in local nodal and distant metastasis, suggesting a local antitumor response to vitamin D in early cancer stages [61].

The function of vitamin D in cancer has been also validated with synthesis of vitamin D analogs, such as MART-10. It has been demonstrated that MART-10 is more potent than 1α,25(OH)2D3 to repress cancer growth and metastasis in undifferentiated thyroid cancer cells. These results require further investigations and *in vivo* study to use MART-10 as potent drug to inhibit anaplastic thyroid cancer cell metastatic potential [62].

Finally, very recently, the effects of calcitriol have been investigated also in a model of thyro-spheres of cell derived from anaplastic carcinomas. The results indicated that calcitriol inhibited proliferation of the anaplastic thyroid carcinoma cells with a more pronounced effect on doxorubicin-resistant cells. Moreover, it reduced the capacity to form stem cell-derived spheres and decreased the size of these spheres, indicating that, also in this model, vitamin D exert a pro-differentiation effect [63].

### Animal models

Dackiw and coworkers implanted in the neck of 4- to 5-weeks-old female SCID mice human thyroid follicular carcinoma derived (WRO). Animals were treated with i.p. injection of 0.75 microg/kg calcitriol or vehicle for 21 day. Average tumor volume presented a 38% reduction (*P* < 0.003) in animals treated with vitamin D. Moreover, tumors excised from calcitriol-treated animals demonstrated signs of differentiation with restoration of thyroglobulin staining not observed in vehicle-treated mice. The results suggested that calcitriol administration can restore p27 accumulation in thyroid carcinoma cells, reducing tumor size and preventing metastatic growth [59]. The results reported by Liu et al. were in the same direction were, demonstrating that calcitriol treatment was able to determine a 50% reduction of tumor weight and a 22% reduction of tumor volume in mice xenografted with tumor cancer cells [64].

### Human studies

The antiproliferative effects of vitamin D have been well characterized *in vitro* and *in vivo* using animal models, and a protective role of vitamin D for non-cutaneous cancer has also been proposed [65]. However, clinical data are still not sufficient to determine whether low circulating vitamin D can be considered a risk factor for cancers, and it is not yet possible to draw definitive conclusions on the effect of vitamin D supplementation on cancer risk [66]. This is true especially for thyroid cancer.

Several reports indicate no significant differences comparing vitamin D levels between cancer patients and healthy controls. In 2010, Laney and coworkers evaluated serum calcium, creatinine, albumin, and 25-hydroxy vitamin D (25-OH-D) in patients with thyroid nodule (42), thyroid cancer in remission (45), and active thyroid cancer (24) and found that serum 25-OH-D was not different between groups. Multivariate regression analysis showed that only a BMI of ≥30 kg/m^2^ can be a good predictor of vitamin D deficiency [67]. No difference in concentrations of 25(OH)D3 between patients with papillary thyroid cancer and patients with HT have been observed by Lizis-Kolus et al. Moreover, in this study, no relationship has been demonstrated between serum 25(OH)D3 and clinical stage of the disease or TSH level in patients with papillary thyroid carcinoma [68]. Also Jonklaas and coworkers demonstrated no associations between vitamin D concentration and thyroid cancer. In addition, serum vitamin D concentrations were not associated with disease stage or other prognostic features [69]. Very recently, Ahn and coworkers evaluated a large group of patients (820) with papillary thyroid cancer. Among those, 795 had insufficient vitamin D levels (<30 ng/mL), however serum vitamin D levels were not associated with disease aggressiveness and poor outcomes [70]. Finally, indirect evidences suggesting no association between vitamin D and thyroid cancers were provided by O’Grady et al. In their report the authors investigated the dietary intake of several micronutrients and were able to establish a relationship only between thyroid cancer and low vitamin C intake, but not with other micronutrients, including vitamin D [71].

Contrasting data suggesting an association between serum vitamin D levels and thyroid cancer have been reported by other groups.

Roskies et al. performed a retrospective cohort study on 212 patients undergoing thyroidectomy. Preoperative 25-hydroxyvitamin D(3) levels were recorded and patients were stratified based on vitamin D status as vitamin D deficiency (VDD) or vitamin D sufficiency (VDS). The comparison of malignancy prevalence between VDD (75%) and VDS (37.5%) indicate a relative risk of 2.0 (*p* =  .03, 95% CI 1.07-2.66) associated to vitamin D deficiency, suggesting that vitamin D may be a modifiable risk factor for thyroid cancer [72]. Similarly, Sahin et al. demonstrated the presence of low serum vitamin D (calcidiol < 20 ng/mL) in 71% of patients with papillary thyroid carcinomas, but only in 59% of controls [73]. Kim et al. studied a total of 548 female patients who underwent total thyroidectomy for PTC. Patients were categorized into four quartiles according to the preoperative serum 25(OH) vitamin D levels, and clinicopathologic features of PTC were analyzed. The results indicated that lower preoperative serum 25(OH) vitamin D levels appear to be associated with poor clinicopathologic features in female patients with PTC [74].

## Conclusion

The majority of the data here reported suggest that vitamin D insufficiency or deficiency may be associated with increased risk of thyroid autoimmunity and that reduced serum concentrations of vitamin D are linked with a major aggressiveness of thyroid cancers. It has to be noted, however, that data are still inconclusive and many papers present contradictory results. Several racial differences have been reported in the scientific literature that may suggest that vitamin D metabolism has also differences linked to ethnicity, which can, at least in part, explain the observed differences.

It is likely that, in order to better understand the role of vitamin D in thyroid disease, several and more extensive prospective studies need to be performed. In addition, in thyroid cancer, it will be necessary to perform long-term supplementation studied to evaluate if higher concentrations of vitamin D may influence the patient outcome.

## Founding

This work has been partially supported with the 2012 founding of the PRIN, from the Italian Ministry for University and Research to PEM.
